# The Contribution of Cortical Lesions to a Composite MRI Scale of Disease Severity in Multiple Sclerosis

**DOI:** 10.3389/fneur.2016.00099

**Published:** 2016-06-29

**Authors:** Fawad Yousuf, Gloria Kim, Shahamat Tauhid, Bonnie I. Glanz, Renxin Chu, Subhash Tummala, Brian C. Healy, Rohit Bakshi

**Affiliations:** ^1^Department of Neurology, Brigham and Women’s Hospital, Harvard Medical School, Boston, MA, USA; ^2^Laboratory for Neuroimaging Research, Brigham and Women’s Hospital, Harvard Medical School, Boston, MA, USA; ^3^Partners Multiple Sclerosis Center, Brigham and Women’s Hospital, Harvard Medical School, Boston, MA, USA; ^4^Department of Radiology, Brigham and Women’s Hospital, Harvard Medical School, Boston, MA, USA

**Keywords:** brain, cortical lesions, multiple sclerosis, cognition, brain atrophy, MRI, physical disability, spinal cord

## Abstract

**Objective:**

To test a new version of the Magnetic Resonance Disease Severity Scale (v.3 = MRDSS3) for multiple sclerosis (MS), incorporating cortical gray matter lesions (CLs) from 3T magnetic resonance imaging (MRI).

**Background:**

MRDSS1 was a cerebral MRI-defined composite scale of MS disease severity combining T2 lesion volume (T2LV), the ratio of T1 to T2LV (T1/T2), and whole brain atrophy [brain parenchymal fraction (BPF)]. MRDSS2 expanded the scale to include cerebral gray matter fraction (GMF) and upper cervical spinal cord area (UCCA). We tested the contribution of CLs to the scale (MRDSS3) in modeling the MRI relationship to clinical status.

**Methods:**

We studied 51 patients [3 clinically isolated syndrome, 43 relapsing-remitting, 5 progressive forms, age (mean ± SD) 40.7 ± 9.1 years, Expanded Disability Status Scale (EDSS) score 1.6 ± 1.7] and 20 normal controls by high-resolution cerebrospinal MRI. CLs required visibility on both fluid-attenuated inversion-recovery (FLAIR) and modified driven equilibrium Fourier transform sequences. The MACFIMS battery defined cognitively impaired (*n* = 18) vs. preserved (*n* = 33) MS subgroups.

**Results:**

EDSS significantly correlated with only BPF, UCCA, MRDSS2, and MRDSS3 (all *p* < 0.05). After adjusting for depressive symptoms, the cognitively impaired group had higher severity of MRI metrics than the cognitively preserved group in regard to only BPF, GMF, T1/T2, MRDSS1, and MRDSS2 (all *p* < 0.05). CL number was not significantly related to EDSS score or cognition status.

**Conclusion:**

CLs from 3T MRI did not appear to improve the validity of the MRDSS. Further studies employing advanced sequences or higher field strengths may show more utility for the incorporation of CLs into composite scales.

## Introduction

Multiple sclerosis (MS) was historically considered a disease targeting CNS white matter, but a growing body of histopathologic and neuroimaging studies have shown involvement of the cerebral cortical and deep gray matter tissue (1–6).

Magnetic resonance imaging (MRI) is an essential tool in clinical MS care including its role in diagnosis and longitudinal monitoring to assess lesions and atrophy of the brain and spinal cord (7, 8). Composite MRI scales are a proposed platform to comprehensively define structural changes related to MS (9–15).

For example, we have developed a Magnetic Resonance Disease Severity Scale, which, in its first version (MRDSS1) (11, 12), included three cerebral measures of MS involvement: T2 hyperintense lesion volume (T2LV, a non-specific measure of overall lesion burden) (16), the ratio of T1 hypointense lesion volume to T2LV (T1/T2, representing the destructive potential of lesions) (15), and normalized whole brain volume (representing whole brain atrophy) (17). MRDSS1 offers more concurrent validity and longitudinal sensitivity than the individual measures on their own (11, 12). In a second version of the scale (MRDSS2), we added measures of cerebral gray matter and spinal cord atrophy, which led to a higher correlation with physical disability than MRDSS1 (13). MRDSS2 also showed a relationship to cognitive dysfunction (13), tested because of the importance of cognition impacting on quality of life (18).

Among the range of newly discovered pathologic changes in MS gray matter, foci of demyelination in the cortical gray matter (cortical lesions, CLs) are of growing interest in representing a core aspect of disease pathogenesis (3, 19, 20). CLs occur early in the MS disease course and are related to physical disability and cognitive impairment (14, 21–32). In this study, we tested the validity of a third version of the MRDSS (MRDSS3) incorporating CLs, compared to the previous versions.

## Materials and Methods

### Subjects

Demographic, clinical, and MRI characteristics of all subjects are summarized in the Table 1. The recruitment methods and inclusion criteria have been described in our previous study (13), from which subjects were drawn for this study, after removing 14 subjects because of technically inadequate MRI scans for CL analysis. As a result, this study included 51 patients with MS and 20 normal controls. Each patient underwent a neurologic examination by an MS specialist, including assessment of expanded disability status scale (EDSS) score (33) and timed 25-foot walk (T25FW) (34). All patients also underwent cognitive testing (see below). Informed consent was obtained from all subjects. This study was approved by our hospital’s institutional review board.

**Table 1:** **Demographic and clinical data**.

	Multiple sclerosis	Normal controls
Number (*n*)	51	20
Age (years)^#^	40.7 ± 9.1 (21.2–55.2)	44.8 ± 6.6 (30.0–53.1)
Women, *n* (%)^^^	35 (69%)	15 (75%)
Disease category, *n* (%)		
Clinically isolated syndrome	3 (5.8%)	–
Relapsing-remitting	43 (84.3%)	–
Secondary progressive	4 (7.8%)	–
Primary progressive	1 (1.9%)	–
Disease duration (years)*	8.3 ± 7.0 (0.2–29.0)	–
EDSS score	1.6 ± 1.7 (0–8.0)	–
T25FW (seconds)	4.9 ± 4.9 (2.9–38.5)	–
Receiving disease-modifying therapy (% of patients)	78.4%	–
MRI variables		
BPF	0.83 ± 0.30 (0.71–0.88)	0.85 ± 0.02 (0.82–0.87)
GMF	0.52 ± 0.30 (0.43–0.57)	0.53 ± 0.02 (0.46–0.56)
T2LV (ml)	13.4 ± 11.9 (2.6–49.3)	0.54 ± 0.67 (0–2.8)
T1/T2	0.42 ± 0.20 (0.11–0.82)	0.31 ± 0.28 (0–0.76)
UCCA (mm^2^)	81.6 ± 9.9 (62.1–103.6)	84.3 ± 10.8 (63.7–109.6)
CLs (count)	2.75 ± 3.0 (0–16)	0

*Data are mean ± SD (range) unless otherwise indicated*.

*EDSS, Expanded Disability Status Scale; T25FW, timed 25-foot walk; BPF, global brain parenchymal fraction; GMF, global cerebral gray matter fraction; T2LV, cerebral hyperintense lesion volume; T1/T2, intra-subject ratio of total cerebral T1 hypointense to T2 hyperintense lesion volume; UCCA, upper cervical spinal cord area; CLs, number of cerebral gray matter cortical lesions*.

Group comparison: ^#^Age: *p* = 0.048, two sample *t*-test; ^^^Sex: *p* = 0.77, Fisher’s exact test; *Time from first symptoms.

### Cognitive Assessment

Neuropsychological evaluation, as detailed in our previous studies (13, 35), employed the Minimal Assessment of Cognitive function in MS (MACFIMS) battery (36). Patients were also evaluated for depressive symptoms using the Center for Epidemiologic Studies Depression (CES-D) scale (37) to adjust for the effect of depression on the relationship between MRI and cognition. Due to the small sample size of the NC group, regression-based norms were acquired using a distinct set of data to control for demographic factors (age, sex, education, and ethnicity), and *T*-scores were calculated (13). We considered a *T*-score of 35 or less as an impairment on any of the MACFIMS elements, permitting subdivision of the MS group into either cognitively impaired (*n* = 18) or cognitively preserved (*n* = 33), based on abnormality of two or more elements of the MACFIMS.

### MRI Acquisition

All subjects underwent MRI on the same scanner (3T Signa; General Electric, Milwaukee, WI, USA) using a consistent acquisition protocol. Brain and cervical spinal cord MRI was performed with the following relevant parameters: brain: coronal 3D modified driven equilibrium Fourier transform (MDEFT) covering the whole head: TR = 7.9 ms, TE = 3.14 ms, flip angle = 15°, slice thickness = 1.6 mm, pixel size = 0.938 × 0.938 mm; axial T2-weighted fast fluid-attenuated inversion-recovery (FLAIR): TR = 9000 ms, TE = 151 ms, TI = 2250 ms, slice thickness = 2 mm, pixel size = 0.976 × 0.976 mm; spinal cord: axial T2-weighted fast spin-echo images of the entire spinal cord: TR = 6117 ms, TE = 110 ms, slice thickness = 3 mm (no inter-slice gaps), pixel size = 0.937 × 0.937 mm. The FLAIR sequence was chosen for the depiction of CLs, based on the effectiveness shown in our previous study (27). We also paired the FLAIR with a high-resolution T1-weighted sequence per our previous strategy to assure accuracy of the identification of CLs and limit false positives (27). The MDEFT was chosen as the T1-weighted sequence, given its effectiveness in gray vs. white structural tissue definition (38) and its high sensitivity to MS lesions, based on our previous work (38).

### MRI Analysis

#### MRI Scan Processing

To facilitate the analysis of CLs using concurrent review of FLAIR and MDEFT scans, the two image sets were brought into the same anatomic plane and matched to the same voxel size by post-processing of the MDEFT scans to match the FLAIR scans. The coronal MDEFT scans were first re-sliced into the axial plane using Jim software (v. 7; Xinapse Systems, West Bergholt, UK, http://www.xinapse.com). The axial MDEFT scans were then co-registered to the native axial FLAIR scans using SPM software (v. 12; Wellcome Department of Cognitive Neurology, London, UK, http://www.fil.ion.ucl.ac.uk/spm/) (39). All MRI analysis was conducted in a blinded fashion without the knowledge of demographic and clinical details.

#### Cortical Lesion Analysis

**Figure 1:**
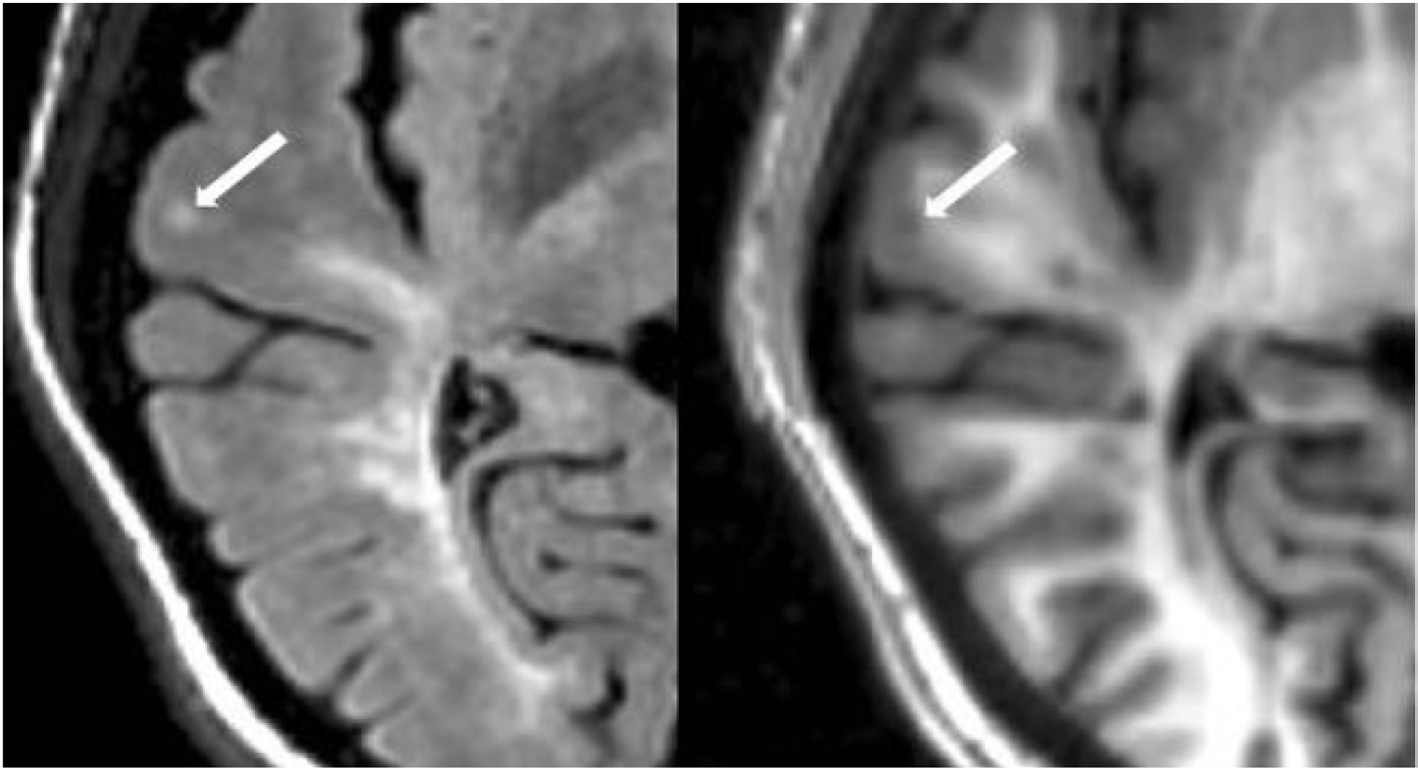
**Fluid-attenuated inversion-recovery scan (left) demonstrating a hyperintense lesion (arrow) that is confirmed on the co-registered modified driven equilibrium Fourier transform scan (right) to show hypointensity (arrow) and involve the cerebral cortex**. This is from a patient with relapsing-remitting MS (34-year-old woman, disease duration = 1 year, Expanded Disability Status Scale score = 1.5).

**Figure 2:**
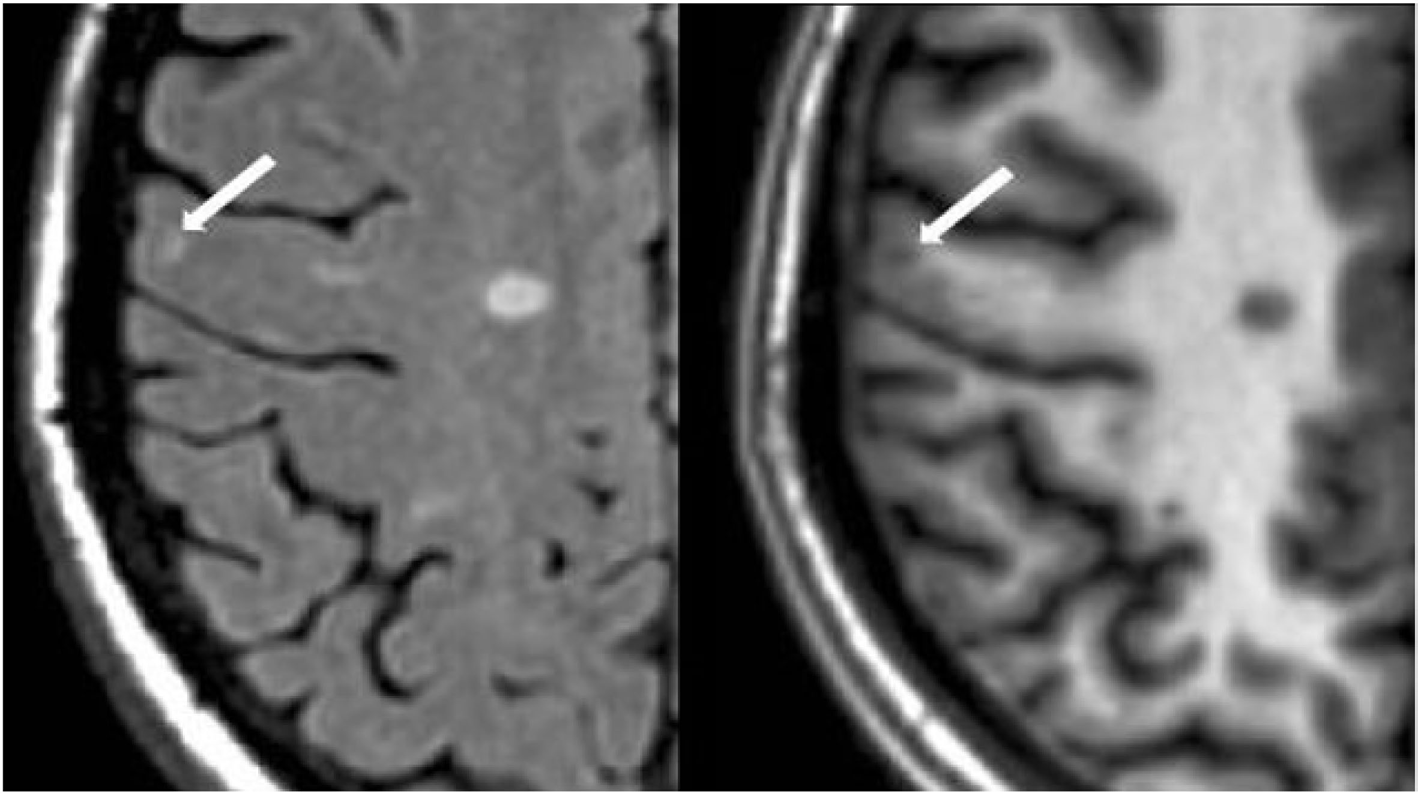
**Fluid-attenuated inversion-recovery scan (left) demonstrating a hyperintense lesion (arrow) that is confirmed on the co-registered modified driven equilibrium Fourier transform scan (right) to show hypointensity (arrow) and involve the cerebral cortex**. This is from a patient with relapsing-remitting MS (29-year-old woman, disease duration = 11 years, Expanded Disability Status Scale score = 2).

**Figure 3:**
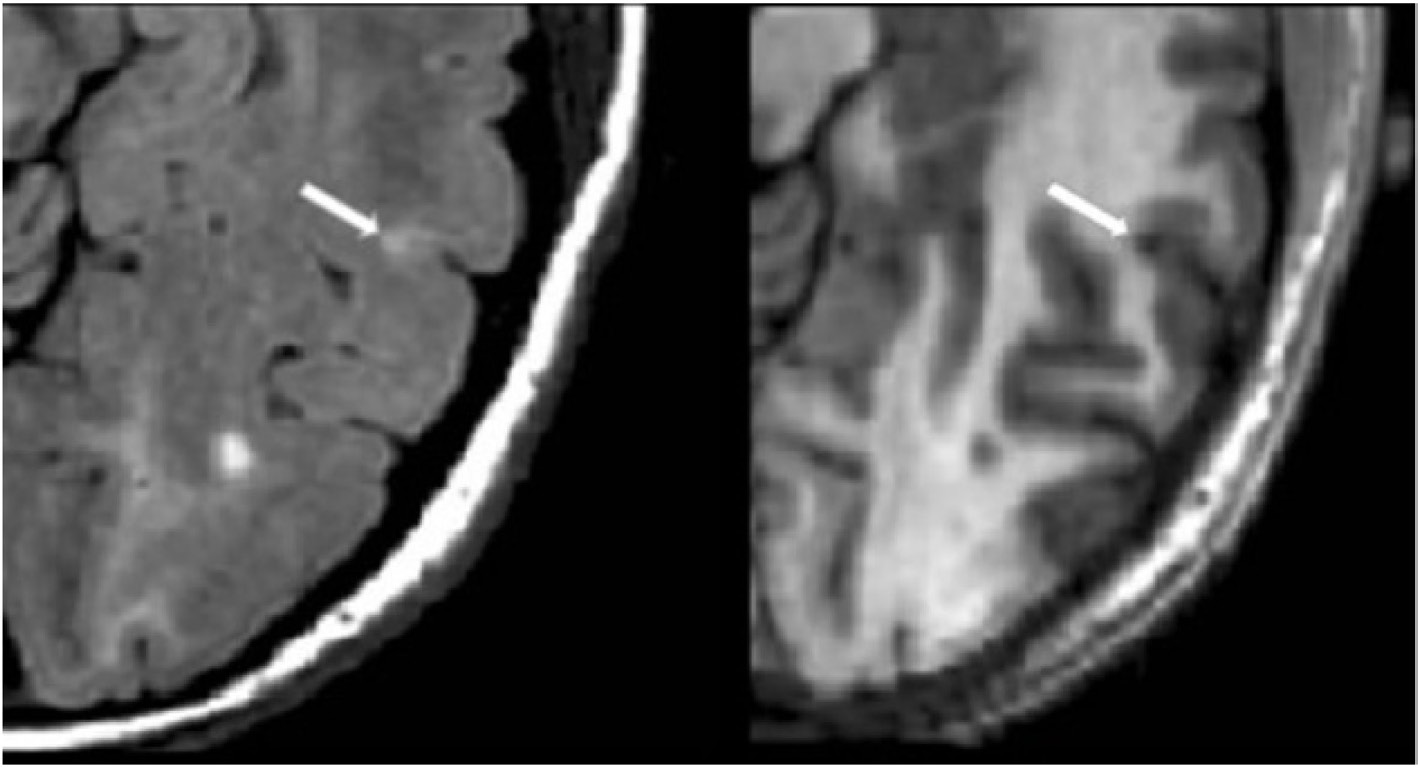
**Fluid-attenuated inversion-recovery scan (left) demonstrating a hyperintense lesion (arrow) that is confirmed on the co-registered modified driven equilibrium Fourier transform scan (right) to show hypointensity (arrow) and involve the cerebral cortex**. This is from a patient with relapsing-remitting MS (42-year-old woman, disease duration = 10 years, Expanded Disability Status Scale score = 0).

**Figure 4:**
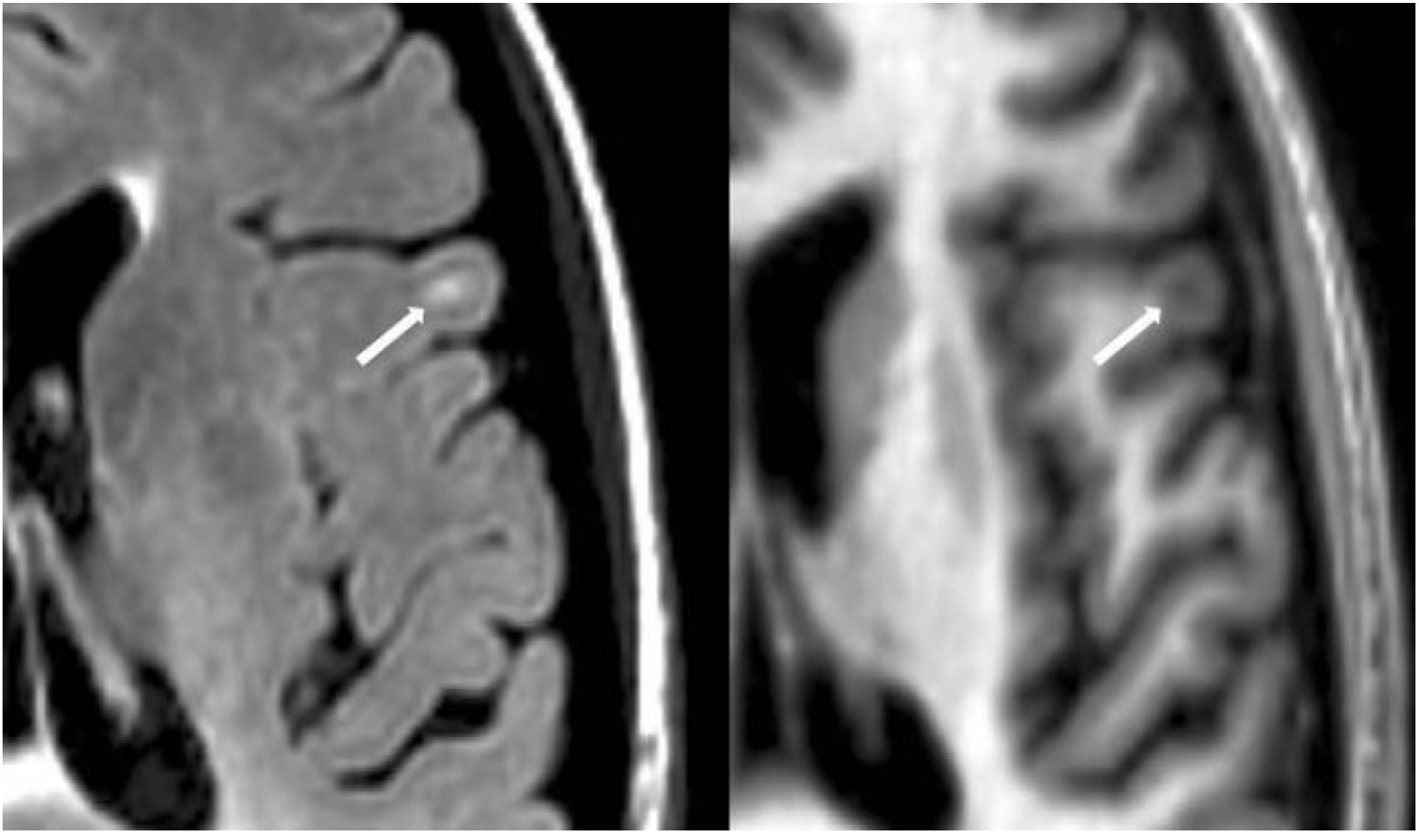
**Fluid-attenuated inversion-recovery scan (left) demonstrating a hyperintense lesion (arrow) that is confirmed on the co-registered modified driven equilibrium Fourier transform scan (right) to show hypointensity (arrow) and involve the cerebral cortex**. This is from a patient with relapsing-remitting MS (40-year-old woman, disease duration = 4 years, Expanded Disability Status Scale score = 3.5).

**Figure 5:**
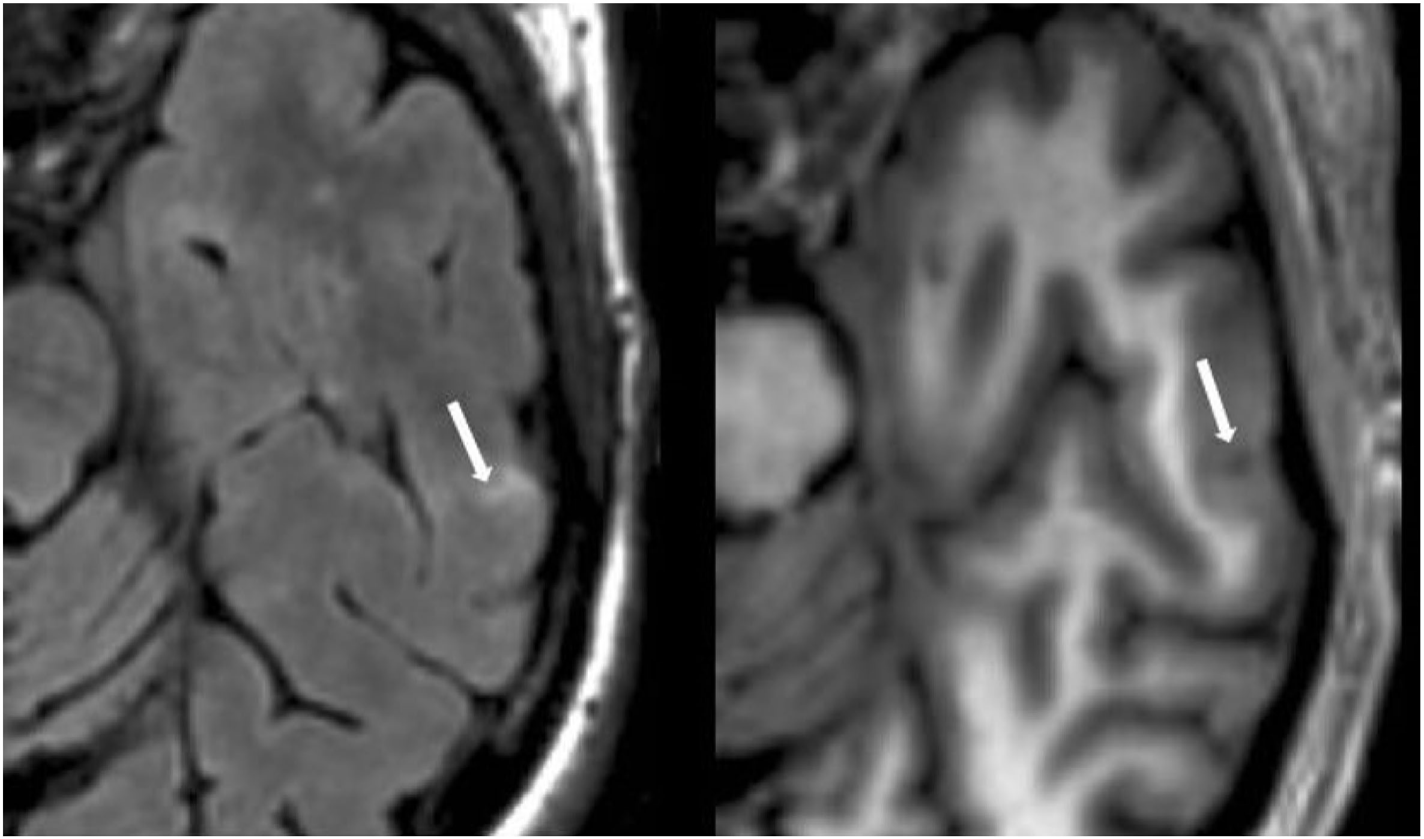
**Fluid-attenuated inversion-recovery scan (left) demonstrating a hyperintense lesion (arrow) that is confirmed on the co-registered modified driven equilibrium Fourier transform scan (right) to show hypointensity (arrow) and involve the cerebral cortex**. This is from a patient with relapsing-remitting MS (44-year-old woman, disease duration = 12 years, Expanded Disability Status Scale score = 3).

The number of CLs was assessed in each case by concurrent review of the co-registered FLAIR and MDEFT sequences in the axial plane in Jim 7. Based on our previously described method (27), CLs were defined as appearing both hyperintense on FLAIR and hypointense on MDEFT images. The lesion was also required to involve at least part of the cerebral cortex on the MDEFT scan. Therefore, juxtacortical lesions, which were seated exclusively in white matter while abutting the cortex, were excluded. No attempt was made to classify CLs into subtypes (40), as this was felt to be technically challenging and beyond the scope of the study. Assessment was made by a reading panel of two trained observers (Fawad Yousuf and Gloria Kim); their findings were confirmed by an experienced observer (Shahamat Tauhid). Any disagreements were evaluated by a senior observer (Rohit Bakshi). Examples of MRI-defined CLs are shown in Figures 1–5. The mean (SD) number of CL in the MS patients was 2.75 (3.0). The specific numbers of CLs found in each subject were 0 lesions (*n* = 4); 1 (*n* = 17), 2 (*n* = 11), 3 (*n* = 9), 4 (*n* = 2), 5 (*n* = 1), 6 (*n* = 4), 9 (*n* = 1), 12 (*n* = 1), and 16 (*n* = 1).

#### Reliability Analysis

Ten randomly chosen MS subjects were reanalyzed to determine intra-rater and inter-rater reliability for the quantification of CLs. Counts of CLs showed high reliability. The mean coefficient of variation was 3.67% within the same rater and 6.47% between the two raters. Regarding intra-class correlation coefficients (ICCs), the intra-rater ICC was 0.98 (model: one-way), and the inter-rater ICC was 0.99 (model: two-way absolute agreement). All three counts agreed for 7 out of the 10 scans, and all scans with 0 or 1 CLs were scored the same. Furthermore, the maximum departure between was 3 lesions, and this was in the subjects with a large number of lesions.

#### Other MRI Analysis

The MRI analysis methodology used to derive lesion volumes, whole brain and gray matter tissue fractions, and upper cervical spinal cord area (UCCA) has been described previously (13). Briefly, cerebral lesion volumes were based on expert identification from FLAIR (T2 hyperintense lesions) and MDEFT (T1 hypointense lesions) followed by semiautomated contouring. Cerebral tissue compartment fractions were derived from MDEFT scans using a validated statistical parametric mapping pipeline (41) to calculate normalized whole brain parenchymal fraction (BPF) and gray matter (GMF) parenchymal fractions. A semiautomated contouring tool (42) served as the foundation for the calculation of UCCA from C2–C5, normalized to cord length, using our highly reliable and validated pipeline on T2 axial images (43).

### Creation of the MRDSS Versions

#### Previous versions of MRDSS (MRDSS1 and MRDSS2)

We have separately described the rationale for the MRDSS1 (11, 12) and MRDSS2 (13). Because of the restricted range of the current MS sample, we relied on d-scores to define the MRDSS versions. We have separately described the method for calculating dMRDSS1 and dMRDSS2 in the present sample based on d-scores (13). dMRDSS1 was calculated as:

dMRDSS1=[−1×dBPF+dlogT2LV+dlogit(T1/T2)]/3 

We defined a d-score to be the observed value minus the mean of the variable in the healthy controls divided by the SD of the variable in the MS sample. For the T2LV and T1/T2, the mean of the healthy controls was calculated only in subjects with non-zero values for each measure. The variables comprising dMRDSS2 differed from dMRDSS1 in two ways: (1) substitution of GMF for BPF and (2) the addition of spinal cord data. dMRDSS2 was calculated as:

dMRDSS2=[−1×dGMF+dlogT2LV+dlogit(T1/T2)  − 1×dUCCA]/4

#### MRDSS3

The variables comprising dMRDSS3 differed from dMRDSS2 only by the addition of counts of CLs. dMRDSS3 was calculated as:

dMRDSS3=[−1×dGMF+dlogT2LV+dlogit(T1/T2)   −1×dUCCA+dCL]/5

The following formula was used to calculate the d-score for CLs:

dCL=(CL number−0)/SDMS (CL number)

### Statistics

Demographic data between groups were compared by using a two sample *t*-test for continuous outcomes and Fisher’s exact test for dichotomous outcomes. Correlations between MRI and neurologic disability in the MS group were assessed using Spearman’s rank correlation coefficients. MRI metrics in the cognition groups were compared by two sample *t*-tests; linear regression was used to adjust for depression (CES-D scores) in these MRI-cognitive comparisons. Correlations among the MRI measures were assessed using Spearman’s rank correlation coefficients. A *p*-value less than 0.05 was considered significant.

## Results

### Correlation between MRI and Disability in the MS Group

We tested the relationship between physical disability (EDSS score) and the three versions of MRDSS along with all the available component MRI measures of brain and spinal cord involvement, as shown in Tables 2 and 4. EDSS showed significant weak-to-moderate correlations, to a similar degree, with BPF, UCCA, dMRDSS2, and dMRDSS3 (all *p* < 0.05).

**Table 2:** **MRI and neurologic disability correlations in the entire multiple sclerosis group (*n* = 51)**.

	Expanded Disability Status Scale	
MRI variable	Spearman’s rho	*p*-value
BPF	−0.306	0.03*
GMF	−0.203	0.15
T2LV	0.001	1.00
T1/T2	0.189	0.18
UCCA	−0.283	0.04*
CLs	−0.058	0.68
dMRDSS1(T2LV, T1/T2, BPF)	0.257	0.07
dMRDSS2 (T2LV, T1/T2, GMF, UCCA)	0.339	0.01*
dMRDSS3 (T2LV, T1/T2, GMF, CL, UCCA)	0.304	0.03*

*BPF, global brain parenchymal fraction; GMF, global cerebral gray matter fraction; T2LV, global cerebral hyperintense lesion volume; T1/T2, intra-subject ratio of total cerebral T1 hypointense to T2 hyperintense lesion volume; UCCA, upper cervical spinal cord area; CLs, number of cerebral gray matter cortical lesions; MRDSS, Magnetic Resonance Disease Severity Scale; MRDSS1, version 1 of the MRDSS; MRDSS2, version 2 of the MRDSS; MRDSS3, (new) version 3 of the MRDSS3. Only MRDSS3 includes CLs*.

*p < 0.05.

**Table 3:** **MRI findings vs. cognitive status in the entire multiple sclerosis group (*n* = 51)**.

MRI variable(s)	Cognitively impaired^a^ (*n* = 18)	Cognitively preserved^a^ (*n* = 33)	*p*-value	Depression-adjusted*p*-value
dBPF	−0.986 ± 1.161	−0.244 ± 0.804	0.02*	0.02*
dGMF	−0.748 ± 1.193	−0.039 ± 0.790	0.03*	0.03*
dT2LV	4.204 ± 1.068	3.665 ± 0.922	0.08	0.08
dT1/T2	0.604 ± 1.114	−0.087 ± 0.854	0.03*	0.03*
dUCCA	0.02 ± 0.976	−0.111 ± 1.025	0.65	0.63
dCL	0.824 ± 0.557	0.981 ± 1.178	0.52	0.52
dMRDSS1 (T2LV, T1/T2, BPF)	1.931 ± 0.868	1.274 ± 0.565	0.008*	0.004*
dMRDSS2 (T2LV, T1/T2, GMF, UCCA)	1.384 ± 0.648	0.932 ± 0.411	0.01*	0.008*
dMRDSS3 (T2LV, T1/T2, GMF, UCCA, CL)	1.272 ± 0.551	0.942 ± 0.482	0.04*	0.06

*^a^Values are presented as mean ± SD; d-scores were calculated by comparing patients with normal controls (see Materials and Methods); BPF, brain parenchymal fraction; GMF, global cerebral gray matter fraction; T2LV, global cerebral T2 hyperintense lesion volume; T1/T2, the ratio of the global cerebral T1 hypointense lesion volume to T2LV in each subject; UCCA, upper cervical spinal cord area; CL, number of cerebral cortical gray matter lesions; dMRDSS1, first version of the Magnetic Resonance Disease Severity Scale; dMRDSS2, second version of MRDSS; dMRDSS3, third (new) version of MRDSS*.

*p < 0.05.

**Table 4:** **MRI and neurologic disability correlations in patients with relapsing-remitting or secondary progressive multiple sclerosis (*n* = 47)**.

	Expanded Disability Status Scale	
MRI variable	Spearman’s rho	*p*-value
BPF	−0.352	0.02*
GMF	−0.254	0.09
T2LV	0.057	0.70
T1/T2	0.129	0.39
UCCA	−0.231	0.12
CLs	−0.053	0.72
dMRDSS1 (T2LV, T1/T2, BPF)	0.275	0.06
dMRDSS2 (T2LV, T1/T2, GMF, UCCA)	0.350	0.02*
dMRDSS3 (T2LV, T1/T2, GMF, CL, UCCA)	0.321	0.03*

*BPF, global brain parenchymal fraction; GMF, global cerebral gray matter fraction; T2LV, global cerebral hyperintense lesion volume; T1/T2, intra-subject ratio of total cerebral T1 hypointense to T2 hyperintense lesion volume; UCCA, upper cervical spinal cord area; CLs, number of cerebral gray matter cortical lesions; MRDSS, Magnetic Resonance Disease Severity Scale; MRDSS1, version 1 of the MRDSS; MRDSS2, version 2 of the MRDSS; MRDSS3, (new) version 3 of the MRDSS3. Only MRDSS3 includes CLs*.

*p < 0.05.

### MRI Findings vs. Cognitive Status in the MS Group

The individual MRI measures as well as the three versions of the MRDSS were compared between the cognitively impaired and the cognitively preserved MS subjects as shown in Tables 3 and 5. BPF, GMF, T1/T2, dMRDSS1, and dMRDSS2 showed higher severity in the cognitively impaired group than the cognitively preserved group both before and after adjusting for depressive symptoms (all *p* < 0.05). dMRDSS3 also showed more severity in the cognitively impaired group as compared to the cognitively preserved before adjusting for depression (*p* = 0.04); however, the significance was lost after adjustment for depression.

### MS Subgroup Analysis

Given the heterogeneous composition of the MS group with regard to clinical course/disease subtype, we also assessed whether the main findings would change with elimination of the four patients with either clinically isolated demyelinating syndromes or primary progressive MS. In the remaining 47 patients (who had either relapsing-remitting or secondary progressive MS), the above results remained similar to the full cohort (Tables 4 and 5).

**Table 5:** **MRI findings vs. cognitive status in patients with relapsing-remitting or secondary progressive multiple sclerosis (*n* = 47)**.

MRI variable(s)	Cognitively impaired^a^ (*n* = 17)	Cognitively preserved^a^ (*n* = 30)	*p*-value	Depression-adjusted*p*-value
dBPF	−1.012 ± 1.191	−0.224 ± 0.831	0.02*	0.02*
dGMF	−0.771 ± 1.225	−0.037 ± 0.815	0.04*	0.04*
dT2LV	4.231 ± 1.095	3.636 ± 0.932	0.04	0.06
dT1/T2	0.577 ± 1.142	−0.095 ± 0.882	0.04*	0.05
dUCCA	0.111 ± 0.924	−0.115 ± 1.070	0.45	0.45
dCL	0.793 ± 0.558	1.023 ± 1.222	0.38	0.40
dMRDSS1 (T2LV, T1/T2, BPF)	1.940 ± 0.894	1.255 ± 0.586	0.009*	0.006*
dMRDSS2 (T2LV, T1/T2, GMF, UCCA)	1.367 ± 0.663	0.923 ± 0.429	0.02*	0.02*
dMRDSS3 (T2LV, T1/T2, GMF, UCCA, CL)	1.252 ± 0.561	0.943 ± 0.504	0.07	0.11

*^a^Values are presented as mean ± SD; d-scores were calculated by comparing patients with normal controls (see Materials and Methods); BPF, brain parenchymal fraction; GMF, global cerebral gray matter fraction; T2LV, global cerebral T2 hyperintense lesion volume; T1/T2, the ratio of the global cerebral T1 hypointense lesion volume to T2LV in each subject; UCCA, upper cervical spinal cord area; CL, number of cerebral cortical gray matter lesions; dMRDSS1, first version of the Magnetic Resonance Disease Severity Scale; dMRDSS2, second version of MRDSS; dMRDSS3, third (new) version of MRDSS*.

*p < 0.05.

### MRI to MRI Correlations

Individual MRI measures were compared with each other for correlations (Table 6). Significant relationships were shown between GMF and BPF (*r* = 0.795, *p* < 0.001) and between T1/T2 and T2LV (*r* = 0.299, *p* = 0.033). CL count was not correlated with any of the other MRI measures (all *p* > 0.05).

**Table 6:** **MRI to MRI correlations in the entire multiple sclerosis group (*n* = 51)**.

	BPF	GMF	T2LV	T1/T2	UCCA
GMF	0.795 (< 0.001)*				
T2	−0.236 (0.095)	−0.149 (0.30)			
T1/T2	−0.236 (0.096)	−0.088 (0.23)	0.299 (0.033)*		
UCCA	0.046 (0.75)	−0.171 (0.23)	0.259 (0.066)	0.075 (0.60)	
CL	−0.096 (0.50)	−0.086 (0.55)	0.136 (0.34)	0.076 (0.60)	−0.022 (0.88)

*Estimated Spearman correlation coefficients (*p*-values) comparing each of the component MRI measures in the MS group; BPF, brain parenchymal fraction; GMF, global cerebral gray matter fraction; T2LV, global cerebral T2 hyperintense lesion volume; T1/T2, the ratio of the global cerebral T1 hypointense lesion volume to T2LV in each subject; CL, number of cerebral cortical gray matter lesions*.

*p < 0.05.

## Discussion

The purpose of our study was to test the role of CLs in adding validity to the MRDSS in defining a relationship between MRI and clinical status in patients with MS. As measures of validity, we assessed physical disability and cognitive impairment. The main findings were that the new version of MRDSS with the addition of CLs (MRDSS3) did not increase the strength of relationships between MRI and clinical status vs. previous versions of MRDSS, in this cross-sectional study.

Our findings were most likely driven by the fact that CLs on their own were not related to EDSS score or cognitive status. There are two previous studies that failed to show a relationship between CL count and EDSS score (44, 45). Whereas, studies that have assessed CL volume have shown closer relationships to EDSS score (22–25, 27). Similarly, previous studies have shown an inconsistent relationship between CL count and cognitive dysfunction, with some studies showing a relationship (14, 25–31, 45), while another study failed to do so (46).

In agreement with our previous study (13), our results emphasized the association between brain/spinal cord atrophy and physical disability and the link between brain atrophy or the destructive potential of brain lesions (T1/T2) and cognitive status. Brain atrophy has long been known to link well to both cognitive impairment and physical disability in MS (47–56). Similarly, spinal cord atrophy has been closely linked to physical disability in patients with MS (13, 42, 57–61).

Furthermore, the cerebral ratio of T1 hypointense to T2 hyperintense lesion load (T1/T2) showed a link to cognitive status in the present study. The persistence of T1 hypointense lesions in patients with MS has long been known to indicate severe destructive pathology corresponding to irreversible demyelination and axonal loss (62, 63). Also, the extent of chronic T1 hypointense lesions in the brain is well correlated with MS related disability (64), whereas an analogous marker of severe CLs is not readily available. However, this is not to diminish the contribution of gray matter involvement to cognition. It is important to note that gray matter atrophy (as assessed by GMF) also linked to cognitive status to a similar extent vs. T1/T2. These results point to the components of both white matter and gray matter pathology in regard to cognitive dysfunction in patients with MS, in keeping with previous observations (65, 66).

We noted some interesting results in the assessment of correlation of the MRI metrics to each other in the MS group. The two cerebral atrophy measures (BPF, GMF) correlated with each other as did the two cerebral lesion measures (T2LV vs. T1/T2). However, spinal cord volume, as estimated by UCCA, did not correlate with any measures of brain involvement. This observation adds to a growing body of evidence suggesting the topographic independence of these two sites of disease activity (59) and the resulting complementary information obtained by considering both brain and spinal cord MRI metrics (13, 67). This divergence is also consistent with previous work showing genetic susceptibility, or immune signatures may confer a specific risk for spinal cord lesions (68, 69). Another interesting finding was the lack of correlation between cerebral lesions and cerebral measures of atrophy. This is a well-known phenomenon when analyzing MS disease course, which may relate to a divergence between inflammatory and neurodegenerative features of the disease in a subset of patients (70). Finally, we also noted a lack of correlation between CLs and measures of cerebral white matter lesions (T2LV or T1/T2). Our findings are in agreement with a previous study (23). When found to be significant in other previous studies, such correlations have remained weak to moderate (21, 22, 24, 25, 27, 28, 30, 44).

However, our study is not without limitations, and the findings should be considered preliminary. First, we would highlight the fact that the average CL count was low in our study as compared to previous studies (21–28, 30, 44, 46, 71). This may relate to several factors inherent in the characteristics of the cohort or technical issues. For example, our patients were mildly disabled, on average, and largely receiving disease-modifying therapy. Our sample was dominated by relapsing-remitting, rather than progressive, forms of the disease. Thus, the degree of cortical involvement may have been limited. The MRI acquisition relied on two types of routine pulse sequences at 3T, without advanced techniques, such as double inversion-recovery (45, 72) and phase-sensitive inversion-recovery (73–75). However, our techniques fell within a clinically feasible high-resolution routine, which should have some value for assessing CLs. We also did not perform 7T field strength MRI to boost the sensitivity in the detection of CLs (25, 76–79). Other strategies, such as increasing the sample size, adding CL volume assessments (22–25, 27, 30), and evaluating CL subtypes (31), may show more utility for improving the validity of the measurement of CLs. It is important to therefore conclude that future studies are necessary to confirm and extend our results.

## Author Contributions

FY, GK, and STauhid: acquisition of data and drafting of the manuscript. BG, RC, and STummala: acquisition of data, editing and approval of the manuscript. BH: statistical analysis and drafting of the manuscript. RB: conceiving the study, obtaining funding, and drafting of the manuscript.

## Conflict of Interest Statement

The authors declare that the research was conducted in the absence of any commercial or financial relationships that could be construed as a potential conflict of interest.
